# Biomechanical Evaluation of Cantilevered Full-Arch Implant-Supported Polymer-Based Hybrid Prostheses: A Digital Image Correlation Study

**DOI:** 10.3390/polym18121457

**Published:** 2026-06-11

**Authors:** Maria Luís Basto, Ana Messias, Maria Augusta Neto, Jack T. Krauser, Fernando Guerra, Ana Martins Amaro

**Affiliations:** 1 University of Coimbra, Centre for Mechanical Engineering, Materials and Processes (CEMMPRE) & Department of Dentistry, Faculty of Medicine, 3000-075 Coimbra, Portugal; marialuis274@hotmail.com (M.L.B.); ana.messias@uc.pt (A.M.); 2 University of Coimbra, Centre for Mechanical Engineering, Materials and Processes (CEMMPRE) & Department of Mechanical Engineering, Faculty of Sciences and Technology, 3030-788 Coimbra, Portugal; augusta.neto@dem.uc.pt; 3 Private Practice Implant Centre of the Palm Beaches, West Palm Beach, FL 33401, USA; jtkrauser@gmail.com; 4 University of Coimbra, Centre for Innovation and Research in Oral Sciences (CIROS) & Department of Dentistry, Faculty of Medicine, 3000-075 Coimbra, Portugal; fguerra@ci.uc.pt

**Keywords:** edentulism, implant-supported prosthesis, full-arch rehabilitation, digital image correlation, poly(methyl methacrylate), poly(ether ether ketone), poly(ether ketone ketone), fiber-reinforced composite

## Abstract

Implant-Supported Fixed Prostheses (ISFPs) have become a common option for the rehabilitation of fully edentulous arches and have traditionally incorporated metallic substructures with ceramic or acrylic veneering. The rapid expansion of CAD/CAM technologies has introduced not only a range of polymer-based materials as alternatives to conventional metallic frameworks but also the possibility of the fabrication of monolithic rehabilitations. However, the evidence regarding the mechanical behavior of monolithic polymer-based full-arch rehabilitations remains limited. This study aimed to evaluate and compare the mechanical performance of monolithic polymer-based complete prostheses under static loading using Digital Image Correlation (DIC). A total of 12 specimens (3 per group) simulating an FP3 maxillary full-arch ISFP supported by four implants were milled from four materials: poly(ether ether ketone) (G1-PEEK), poly(ether ketone ketone) (G2-PEKK), poly(methyl methacrylate) (G3-PMMA), and fiber-reinforced composite (G4-FRC). All specimens were subjected to static loading up to 200 N at the incisors region, corresponding to the anterior unsupported span, and at the occlusal surface of the molars, corresponding to the most distal portion of the cantilever, using a universal testing machine. Full-field vertical displacement and strain distributions (principal tensile, compressive, and von Mises) were acquired through a stereo DIC system and analyzed using a Linear Mixed-Effects Model with Tukey’s HSD post hoc comparisons (α = 0.05). All prostheses withstood the applied load without macroscopic failure. G3-PMMA exhibited the highest vertical displacement, exceeding 1000 µm in the anterior span and 1500 µm in the cantilever region, along with the greatest strain concentrations, particularly at the interproximal embrasures distal to the terminal abutment. G1-PEEK provided the lowest displacement in the anterior span. G4-FRC presented displacements similar to G1-PEEK and G2-PEKK at the distal cantilever, but the lowest tensile strains and the most homogeneous strain dissipation in both loading at the anterior unsupported span and distal cantilever. This indicated that the biomechanical performance of full-arch ISFPs is highly influenced by the polymer used. PEEK, PEKK, and FRC appear as promising alternatives to PMMA for monolithic full-arch rehabilitations.

## 1. Introduction

Edentulism is an irreversible condition, classified as a disability by the World Health Organization (WHO), and may be defined as the absence of natural dentition in the oral cavity. It has a substantial and obvious impact on the oral health and overall well-being, affecting the physical, social, and psychological dimensions of patients’ lives [1,2].

For more than a century, the standard treatment for fully edentulous patients consisted of conventional complete dentures. However, implant-retained overdentures or implant-supported fixed prostheses (ISFPs) have become the preferred treatment options to overcome the well-known limitations of conventional prostheses, such as reduced masticatory efficiency, speech difficulties, decreased occlusal force, impaired oral sensory feedback, and compromised patient comfort. This, obviously, is dependent on individual patient factors, including economic status, systemic health, health habits, and bone availability [1,3,4].

The long-term success and survival of the ISFPs have been well documented in the literature. Chrcanovic et al. [5] reported survival rates of 93.3% after 10 years and 87.1% after 20 years. Nevertheless, treatment outcomes depend on several variables, such as the number and distribution of implants and the extension of the cantilever (the unsupported portion extending posterior to the last implant designed to increase the occlusal contact area), the prosthesis design and material [4,5,6].

Regarding the number and distribution of implants, the literature provides strong evidence that four implants represent the minimum recommended number for rehabilitation with an ISFP, increasing to five or six whenever anatomical features and patient-related factors allow. A greater number of implants may reduce the length of unsupported sections, either in inter-implant spans or cantilever regions, and improve occlusal load distribution. Clinicians should aim for symmetrical implant placement, maximizing the spread between the anterior and posterior regions while ensuring that the conditions obtained provide the possibility of achieving passive fit and adequate prosthetic rigidity [7,8,9].

When a four-implant configuration is used, the prosthetic components and, subsequently, the peri-implant bone may be subjected to deleterious stresses resulting from the flexure forces generated by cantilever extensions. Excessive cantilever length has been associated with an increased risk of both biological and mechanical complications, with the incidence of such complications in ISFPs reaching 20.3% at 5 years, more than double that observed in non-cantilever ones [4,10,11,12].

Several other studies have shown that the use of distal cantilevers generates the highest stress concentrations, with excessive strains at implant supports, increased lever-arm effects, and consequent bending moments [13,14,15]. A second critical region often arises from the need to maximize anteroposterior spread through posterior implant positioning, generating a wide anterior unsupported span. This configuration results in increased inter-implant distance between the two anterior implants and may lead to midline prosthetic deformation, particularly under high incisal loading and in round configurations [4,10,11].

The prosthetic material also plays a critical role in the success and overall durability of the restoration, as it directly influences load-bearing capacity and the transmission of stress to the supporting implants, peri-implant tissues, and surrounding bone during masticatory cycles, functional loading, and eventual parafunctional activities. The forces transferred from the ISFPs to the implants and subsequently to the bone-implant interface may contribute to both mechanical and biological complications [4,16,17]. Traditionally, metallic materials such as titanium and cobalt-chromium alloys have been considered the gold standard for the framework of ISFPs because of their biocompatibility and favorable mechanical properties. However, concerns have been raised regarding not only their high elastic modulus and rigidity, which may increase stress concentration at the implant-bone interface, potentially contributing to peri-implant bone loss or implant failure, but also regarding their biomechanical degradation due to fatigue and corrosion [18,19,20,21].

In response to these limitations, several non-metallic alternatives have been investigated to obtain prosthetic structures with lower elastic modulus, reduced weight, lower cost, and improved stress distribution to the implant-bone interface. With the rapid expansion of CAD/CAM technologies and 3D printing in dentistry, a broad range of polymer-based materials has emerged as an alternative to metals and metal alloys for ISFP and other fixed restorations [19,22].

Materials such as poly(methyl methacrylate) (PMMA), poly(ether ether ketone) (PEEK), poly(ether ketone ketone) (PEKK), and fiber-reinforced composites (FRC) can be milled into monolithic structures and subsequently characterized as monolithic polymer-based prostheses. This approach may reduce the risk of fracture associated with ISFP that use frameworks and veneering materials since the whole volume of the teeth is manufactured as an integral part of the prostheses rather than being bonded to the framework. In addition, this digital workflow is generally less time-consuming, requires reduced laboratory intervention, and may also reduce clinical adjustments because of the greater precision reported for digital manufacturing. However, evidence regarding the long-term clinical success and mechanical behavior of monolithic polymer-based prostheses remains limited, and further studies are needed to support their potential advantages [23,24,25].

PMMA is widely used in several areas of dentistry, particularly in prosthodontics. In the context of ISFPs, PMMA has traditionally served as the reference material for interim provisional restorations. As a definitive treatment option, however, in vivo studies reporting clinical and patient-centered outcomes remain limited. In addition, the inherent limitations of PMMA include relatively low mechanical strength, surface degradation, and difficulties in achieving a highly accurate fit, which may result in a greater flexure under load, associated deformation, and stress concentration within the prosthesis. Nevertheless, the limited number of available clinical studies and case reports suggests that milled PMMA full-arch implant-supported prostheses may represent a viable restoration option in the short term, with acceptable success rates and favorable patient satisfaction, particularly because they are straightforward, esthetic, time-efficient, and cost-efficient [26,27].

Accordingly, in the search for an ideal prosthetic material combining biocompatibility, adequate mechanical properties, favorable esthetics, and reduced transmission of harmful forces to the supporting bone and soft tissues, new polymeric materials have been introduced into implant prosthodontics. These include high-performance engineering polymers such as PEEK and PEKK, as well as FRCs. In addition to allowing for CAD/CAM milling, these materials exhibit improved mechanical properties, including higher elastic modulus and greater flexural strength when compared with conventional polymers such as PMMA. Such characteristics may provide greater structural rigidity and consequently reduce displacement and deformation under functional loading.

PEEK and PEKK are non-toxic, inert, and radiolucent polymers with high biocompatibility, water resistance, low plaque affinity, and low weight, and they have been applied in several areas of dentistry. Their broad interest in dentistry is partly related to their mechanical and physical properties, which resemble those of human bone and dentin, particularly because their Young’s modulus (PEEK: ~4.4 GPa; PEKK: ~3.4 GPa) is closer to bone than that of metallic materials [1,11,20]. Although in theory, these materials exhibit favorable biocompatibility and have been proposed as suitable alternatives to conventional materials, in vivo evidence remains scarce. Available in vitro studies investigating the mechanical behavior of these polymers from the poly(aryl ether ketone) (PAEK) family indicate a low shock-absorbing effect, potentially increasing stress transfer to dental implants and the peri-implant bone [3,14,28].

Another alternative to metallic materials is the new generation of CAD-CAM fiber-reinforced composite resins (FRCs). These materials contain a higher concentration of multidirectional glass fibers than conventional composite resins and have, therefore, been increasingly applied in prosthodontics because of their capacity to absorb masticatory impact and dissipate functional energy. In addition, they present low elastic modulus, high flexural strength (approximately 420 MPa), and reduced cost, weight, and density [4,19].

Although several recent studies have investigated the mechanical behavior of these materials, most have focused on implant-supported fixed prosthesis (ISFP) frameworks [1,4,11]. Consequently, there is limited evidence regarding a monolithic approach using an FP3-like design, characterized by prostheses geometry and volume intended to replace not only missing teeth, but also lost alveolar bone and peri-implant soft tissues. Therefore, a better understanding is needed of how PEEK, PEKK, and FRCs behave under loading in comparison with PMMA, particularly in wide-span restorations and long-cantilever scenarios.

Accordingly, the present study aimed to evaluate and compare the mechanical performance of milled complete prostheses under static loading in cantilever and unsupported-span conditions using Digital Image Correlation (DIC). The findings of this study may provide clinically relevant guidance for material selection in full-arch implant-supported rehabilitations.

The null hypothesis of the study was that no significant differences would be observed among the tested materials regarding vertical displacement and maximum strain values.

## 2. Materials and Methods

### 2.1. Models Preparation

Complete prosthetic models for full arch maxillary ISFPs supported on 4 implants used in the experiment were fabricated by milling of blocks of four different polymers: poly(ether ether ketone) (G1-PEEK), poly(ether ketone ketone) (G2-PEKK), poly(methyl metacrilate) (G3-PMMA) and fibre-reinforced composite (G4-FRC). The manufacturer’s catalogues indicate that G1-PEEK samples (JUVORA™ PEEK, Institut Strauman AG, Basel, Switzerland) present 165 ± 10 MPa flexural strength, G2-PEKK (Pekkton^®^ ivory, Cendres+Metaux, Biel, Switzerland) specimen have 5.0 GPa flexural modulus and G4-FRC (Trilor™ Disk, Bioloren S.r.l., Saronno, Italy) 540 MPa flexural strength and 26 GPa flexural modulus.

**Figure 1:**
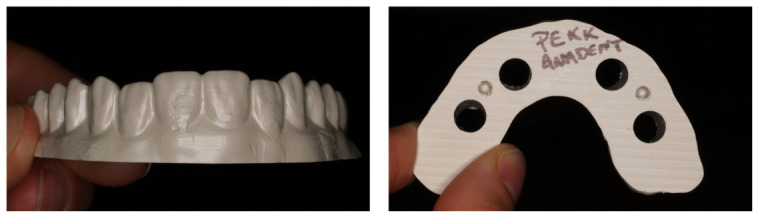
Sample of G2-PEKK showing the four holes in the structure corresponding to the implant position.

For each group, three specimens were produced per group, totaling 12 complete ISFPs, with four holes in the structure, corresponding to the position of the implants (Figure 1).

Before experimental testing, all specimens were air-sprayed with airbrush pro-color ink (Hansa Airbrush, Norderstedt, Germany), allowing the surface to be coated with the non-repetitive, isotropic speckle pattern required for full-field displacements and deformation measurements using the Digital Image Correlation (DIC) method.

### 2.2. Experimental Setup

**Figure 2:**
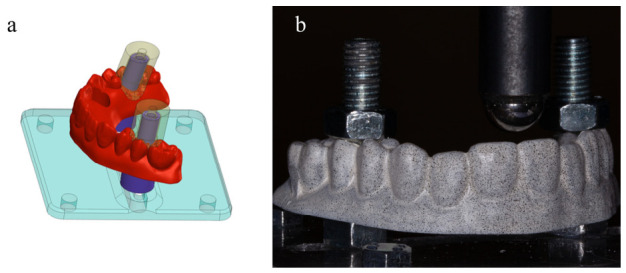
Assembly of the ISFPs in the metallic support for testing in the universal testing machine. (**a**) Digital planning of the setup (metallic plate and screws) for loading the anterior portion of the ISFP. (**b**) Coated sample fixed to the universal testing machine, aligned with the loading sphere.

For mechanical testing, the complete prostheses were fixed to a previously milled metallic base using M7 screws and nuts, as shown in Figure 2. For testing the anterior span, the screws were applied over the holes made.

This assembly was then attached and fixed to the universal testing machine (AG-I Shimadzu^®^; Shimadzu Scientific Instruments, Columbia, MD, USA), with a maximum load capacity of 5 kN. The machine was controlled through dedicated software, TRAPEZIUM X (Version 1.5.1, Shimadzu^®^ Corporation, Columbia, MD, USA), in which the compression cycles were programmed. The complete prostheses were subjected to static load-bearing capacity tests, with load applied through a spherical contact surface located at the tip of the loading screw (Figure 2). Two loading points were evaluated: the incisor region and the molar region. During loading of the incisor’s region, only the posterior fixation screws remained in place, whereas during loading of the molar region, only the anterior fixation screws were maintained. Load was applied increasingly from 0 to a maximum of 200 N at a crosshead speed of 0.5 mm/min. The maximum load of 200 N was selected based on both clinical relevance and the requirements of the DIC methodology. Although maximum voluntary bite forces in the posterior region can exceed 600 N, functional masticatory forces during normal chewing activity are commonly reported within the range of 100 to 300 N, framing 200 N within physiological functional loading conditions. Furthermore, the objective of the study was to perform a non-destructive evaluation of the strain distribution and kinematic behavior of the prostheses. The application of higher loads could have induced plastic deformation or premature failure in materials with lower mechanical resistance, particularly PMMA, thereby compromising the ability to perform standardized comparisons among the tested materials. By limiting the applied load to 200 N, all specimens were maintained within their elastic phase, allowing reliable assessment and precise comparison of strain distribution patterns under clinically relevant loading conditions.

For the capture of images for digital image correlation, two high-speed cameras (Stingray F-504B ASG, Allied Vision, Andover, MA, USA), with a maximum resolution of 1624 × 1224 pixels (4.4 mm pixel size) and a maximum frame rate of 19 frames per second, were mounted on a tripod at approximately 1 m away from the specimens. The cameras were also positioned symmetrically relative to the specimens with a stereo angle (angle between cameras) below 45° to preserve constant magnification and provide synchronized image acquisition throughout the experiments, as recommended by the manufacturer. The cameras were connected to a computer running VIC-3D 2012 software (Correlated Solutions^TM^, Columbia, SC, USA), which was used for image acquisition, processing, and analysis. In this study, a small, dense, irregular-shaped speckle pattern of approximately 5 × 5 pixels was applied to the surface of every specimen, and a subset size of 29 was considered for analysis. The light source was strategically positioned to ensure correct illumination of the speckle pattern and thus minimize reflections and potential sources of error.

Before loading the prostheses, the stereo system was calibrated. This procedure was performed by acquiring several images using a 30–45 mm field of view target with a 12 × 9 dot grid with 4 mm spacing between dots. This process enabled system calibration and established the relationship between the camera coordinates and the real coordinates of the model, allowing the transformation algorithm to convert deformation into displacement values. After processing the calibration images, the software generated an error score of less than 0.1 pixels for all tests, representing the difference between the real and calculated positions. These low error values indicate acceptable calibration accuracy and the absence of systematic errors in the present study.

After loading and image acquisition, a region of interest was defined in the speckle-pattern images for which full-field displacements along the transformed U (lateral), V (vertical), and W (anterior–posterior) axes and full-field strain data (Ɛ1 (principal tensile strains), Ɛ2 (principal compressive strains), and ƐvM (von Mises strains) were extracted from the DIC software.

To account for clustering of the full-field DIC data and the inherent spatial correlation of thousands of data nodes nested within individual physical specimens, a Linear Mixed-Effects Model (LMM) was employed, designating material (G1-PEEK, G2-PEKK, G3-PMMA, G4-FRC) as the fixed effect, and the individual specimen as the random effect.

Descriptive statistics for the global biomechanical behavior of each material were computed and reported as Estimated Marginal Means (EMMs) with 95% Confidence Intervals (CIs). To characterize the maximum localized deformation while filtering out potential optical noise artifacts inherent to DIC surface tracking, extreme values were reported utilizing the 99th percentile for maximum tensile and von Mises strains and the 1st percentile for maximum compressive strains and vertical displacement. Post hoc pairwise comparisons between the restorative materials were conducted using Tukey’s HSD correction. All statistical analyses were executed in R software (RStudio 2026.01.0+392 “Apple Blossom” Release), utilizing the **lme4** package for mixed modeling and the **emmeans** package for marginal means calculation. The significance level was established at α = 0.05.

## 3. Results

All monolithic prostheses resisted the applied load at both the anterior span and the posterior cantilever portion without exhibiting visible signs of failure, such as fracture lines or other cracks.

**Figure 3:**
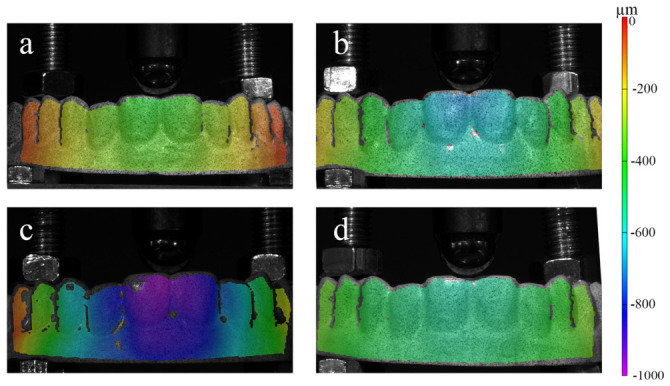
Graphical representation of the full-field vertical displacements determined for the surface of the anterior unsupported span of the complete prostheses of (**a**) G1-PEEK, (**b**) G2-PEKK, (**c**) G3-PMMA, and (**d**) G4-FRC at 200 N load. Same color scale for all images. Values in μm.

Kinematic analysis revealed that displacement occurred predominantly in the direction of the applied load (vertical) with negligible horizontal movements (lateral or anterior–posterior). The full-field distribution of vertical displacements under a 200 N load in the anterior region of the prostheses is presented in Figure 3. The images indicate that G3-PMMA presented the highest vertical displacement, deflecting in excess of 1000 µm at the load application area in the incisor’s region. In contrast, the remaining groups demonstrated lower and relatively similar vertical displacement values, ranging from approximately 400 to 600 µm in the same region.

**Figure 4:**
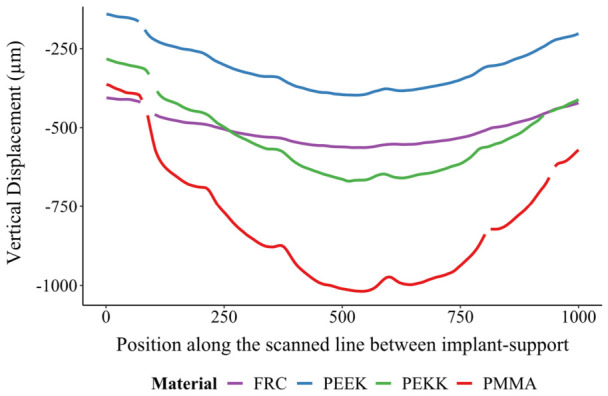
Distribution of vertical displacement (mm) along an inspection line extending from 1st premolar to 1st premolar, at the anterior unsupported span, with loading applied at the incisor’s region.

A more detailed analysis of the prosthesis displacement is shown in Figure 4, which illustrates the profile of vertical displacement along the unsupported span, based on an inspection line extending between the first premolars. All materials mimic the flexural behavior of a beam supported at both ends under a central load. G3-PMMA displayed a more pronounced parabolic curve, indicating greater peak deformation compared with all the other groups. The flattest and most uniform profiles were observed for G1-PEEK or G4-FRC, with G1-PEEK presenting the lowest maximum vertical displacement. The peak values of each variable studied are detailed in Table 1.

**Figure 5:**
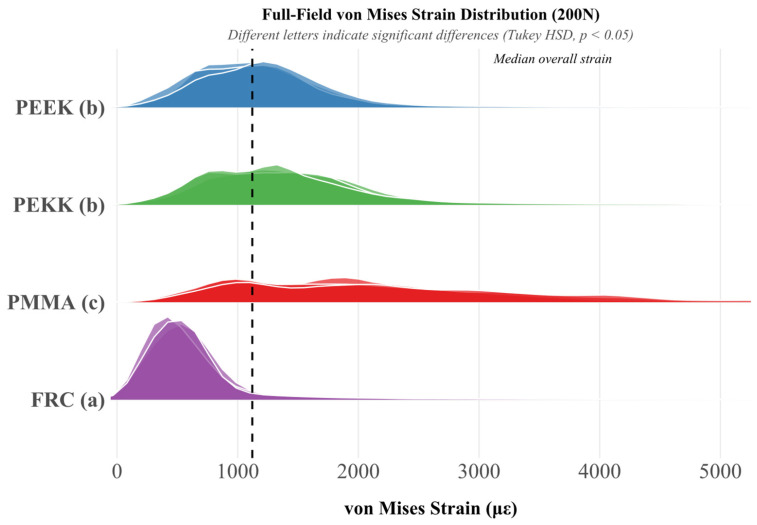
Probability density distributions (ridgeline plots) derived from the full field distribution of von Mises strain (με) at the anterior unsupported span. Horizontal axis indicates the magnitude of the strain, while the height of the curve represents the relative frequency of that specific strain value occurring across the anterior unsupported span. The dashed vertical line represents the overall global median. A higher peak denotes a greater concentration of the framework experiencing that specific strain, whereas an elongated tail indicates localized regions of extreme deformation. Three distributions per material, corresponding to the tested specimen. Similar letters between brackets indicate groups that do not differ.

Figure 5 details the distribution of the von Mises strains in the anterior span, where the vertical dashed line represents the overall median strain value across all samples. A total of 89.7% of the nodes in G4-FRC were below this mean value, whereas a substantial proportion (77.7%) of nodes in G3-PMMA exceeded it, indicating excessive surface deformation. G1-PEEK and G2-PEKK exhibited a more balanced distribution profile, with 52.3% and 63.4% of nodes above the median line, respectively.

**Figure 6:**
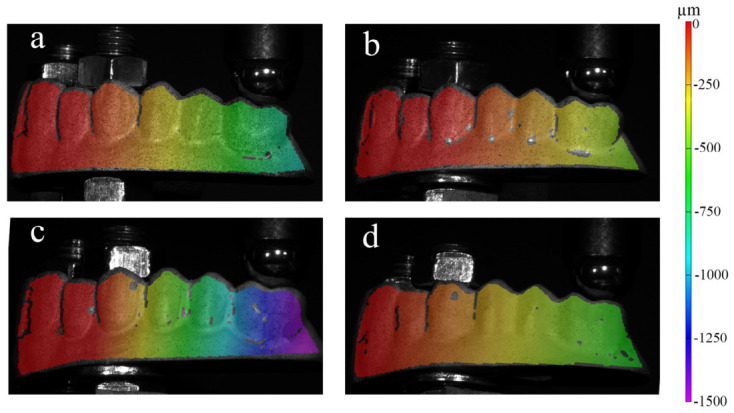
Graphical representation of the vertical displacements determined for the surface of the cantilever portion of the complete prostheses of (**a**) G1-PEEK, (**b**) G2-PEKK, (**c**) G3-PMMA, and (**d**) G4-FRC at 200 N load. Same color scale for all images. Values in μm.

**Figure 7:**
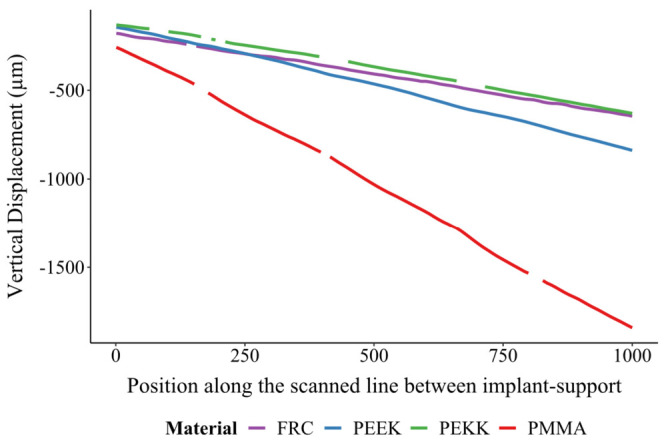
Distribution of vertical displacement (mm) along an inspection line extending from the point of fixture to the 1st molar, at the cantilever portion, with loading applied over the distal cusp of the molar.

In the cantilever portion of the prostheses, displacement occurred vertically, linearly increasing from the fixed mesial support toward the distal free end of the prosthetic flange of all materials (Figure 6). While G1-PEEK, G2-PEKK, and G4-FRC exhibited similar vertical displacement values, not exceeding 600 µm, G3-PMMA demonstrated a two-fold increase in structural deflection, with the most distal segment displacing in excess of 1500 µm under 200 N load, as identified by nodes in purple. A more detailed analysis of the prosthesis displacement is shown in Figure 7, which illustrates the profile of vertical displacement along the distal cantilever, based on an inspection line extending between the first incisor and the first molar.

The quantitative displacement and strain values for the cantilever extension are summarized in Table 2.

**Figure 8:**
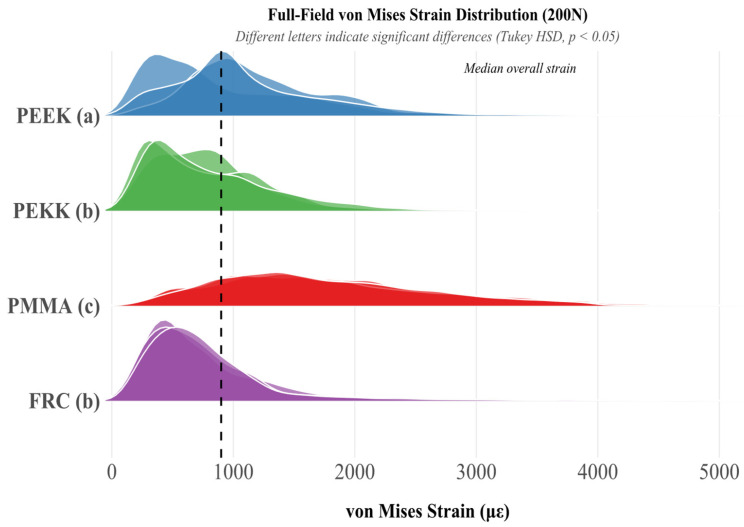
Probability density distributions (ridgeline plots) derived from the full field distribution of von Mises strain (με) at the cantilever region (molars region). Horizontal axis indicates the magnitude of the strain, while the height of the curve represents the relative frequency of that specific strain value occurring across the cantilever. The dashed vertical line represents the overall global median. A higher peak denotes a greater concentration of the framework experiencing that specific strain, whereas an elongated tail indicates localized regions of extreme deformation. Three distributions per material, corresponding to the tested specimen. Similar letters between brackets indicate groups that do not differ.

The full-field von Mises strain distribution in the cantilever region of all four polymeric materials is presented in Figure 8, and further corroborates the structural limitations of G3-PMMA. This material demonstrated the highest percentage of nodes above the overall median strain value (83.2%), followed by G1-PEEK (54.4%). Notably, G2-PEKK and G4-FRC demonstrated highly efficient and structurally comparable strain dissipation. Post hoc statistical analysis assigned both materials to the same significance grouping (letter b), indicating no statistically significant difference in their global strain distributions, with 35.1% and 27.2% of nodes above the global median value, respectively.

**Figure 9:**
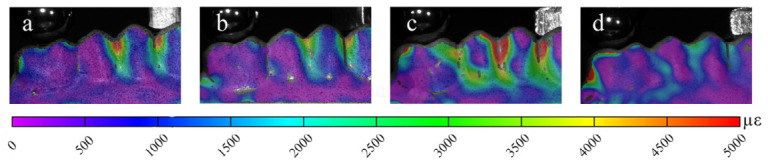
Graphical representation of full-field principal tensile strains (Ɛ1) determined for the surface of the cantilever portion of (**a**) G1-PEEK, (**b**) G2-PEKK, (**c**) G3-PMMA and (**d**) G4-FRC at 200 N. Same color scale for all images. Values in µε.

Figure 9 illustrates the full-field distribution of principal tensile strains across the posterior cantilever segment. The strain maps highlight severe, localized deformation within G3-PMMA, specifically concentrated at the interproximal embrasures extending distally from the terminal abutment (canine-premolar junction) to the premolar-molar region. These critical tensile concentrations denote a high risk of flexural fatigue and bending-induced fracture. Conversely, the G4-FRC group demonstrated a highly efficient dissipation of tensile forces, yielding the lowest absolute tensile strains and the most balanced structural distribution under unilateral loading.

## 4. Discussion

The present study evaluated displacement and strain distribution on the surface of complete ISFPs fabricated from four different polymeric materials, using DIC, under progressively increasing load ranging from 0 to 200 N. The results revealed significant differences among the tested materials in terms of both displacement and von Mises strain values. These findings suggest that the material in which the complete prostheses are milled plays a critical role in load distribution and overall structural stability under functional loading conditions.

As previously described (Section 3), all tested prostheses were able to withstand the applied load without any macroscopic failure, indicating that all polymeric materials exhibited sufficient structural integrity under loads up to 200 N. However, this apparent mechanical viability does not mean that these materials have optimal biomechanical properties and performance, as the obtained results demonstrated clear differences in displacement and strain behavior among all.

The displacement in both simulated situations was mainly vertical, with negligible horizontal movements, which means the experimental setup, particularly the fixation system, was effective. In the anterior unsupported span, all materials exhibited deformation consistent with beam-like flexural behavior under incisor region loading. However, G3-PMMA showed significantly greater displacement (around 1000 µm), while the remaining materials showed a more limited one (range 400 to 600 µm). This pronounced deformation in PMMA is consistent with its lower elastic modulus, resulting in reduced resistance to bending under load [1]. G1-PEEK and G4-FRC, demonstrated the lowest displacement values and more linear and uniform displacement profiles, indicating superior structural rigidity and improved load distribution across the anterior span, which are according with [1], associated with higher elastic modulus and flexural strength.

The von Mises strain analysis supports the above statement, with PMMA, in the anterior span, showing a high proportion of the nodes above the median strain value, reflecting the highest surface deformation, which is consistent with the strain values listed in Table 1. On the other hand, FRC showed a lower proportion of the nodes exceeding that same median value, being the material with the lowest values, corresponding to a more favorable strain distribution. G1-PEEK and G2-PEKK demonstrated an intermediate and more balanced strain distribution, which indicate a compromise between structural rigidity and energy absorption capacity.

In the cantilever region, all materials showed a linear increase in vertical displacement values from the mesial support toward the distal free end, as expected under a cantilever loading situation (Figure 4 and Figure 5). Once again, G3-PMMA showed the highest displacement values, reaching over 1500 μm, approximately twice those observed for the other materials.

This trend was again confirmed with the von Mises strain values for the cantilever portion. PMMA once more showed the highest proportion of the nodes exceeding the global median strain, which restates greater deformation concentration under loading. In contrast, G2-PEKK and G4-FRC exhibited the lowest strain values, and therefore, the most favorable strain distribution, with only a smaller percentage of the nodes above the median line and no significant difference between the two. G1-PEEK showed intermediate behavior and strain distribution, with higher values than PEKK and FRC but still much lower than PMMA, consistent with the findings in the anterior span situation.

Overall, these results indicate that materials with higher elastic modulus within the group evaluated in, and thus more stiffness, such as PEEK, PEKK, and FRC, are less susceptible to displacement and deformation, with a more favorable strain distribution compared to PMMA. Although PMMA remains a widely used material for provisional restorations and interim prostheses, its mechanical behavior under loading, especially when it comes to critical configurations such as the ones simulated in this study (anterior unsupported span and distal cantilever portion), suggests limitations for long-term use in milled definitive full-arch rehabilitations. In contrast, high-performance polymers and fiber-reinforced composites appear to offer biomechanical advantages that may translate into a superior clinical performance. Nevertheless, these materials differ substantially from conventional framework materials such as titanium (elastic modulus ≈ 110 GPa) and zirconia (elastic modulus > 200 GPa), which exhibit considerably greater rigidity and structural durability. While the high stiffness of these conventional materials promotes efficient load transfer, it may also result in a more direct transmission of stresses to the implant components and surrounding crestal bone. The lower elastic modulus of PEEK, PEKK, and FRC (approximately 3 to 15 GPa) provides greater shock-absorbing capacity, attenuating peak occlusal forces and promoting a more favorable stress distribution. This damping effect may be particularly advantageous in clinical scenarios where stress dissipation is desirable, such as immediate loading protocols, full-arch rehabilitations opposing implant-supported ceramic restorations, and patients with parafunctional habits. Although these polymer-based materials cannot match the rigidity of titanium or zirconia, their biomechanical behavior may represent a beneficial alternative in selected clinical cases.

Nonetheless, these findings should be interpreted with caution due to the in vitro nature of the present study. The quasi-static loading conditions employed in the present study do not fully reproduce the complex cyclic and multidirectional masticatory forces encountered in the oral environment. While the application of quasi-static loading represents an important limitation of this investigation, it also provides a highly controlled and reproducible baseline for assessing the biomechanical behavior of restorative materials.

The evaluation of the full-field principal tensile strains under 200 N load allowed the identification of the exact regions that exhibited the greatest deformation and strain concentration, which are recognized as critical sites for stress accumulation and potential fatigue micro-crack initiation in cyclic loading scenarios. Consequently, although the present DIC analysis cannot predict absolute clinical longevity or long-term survival rates, it provides valuable insights into the relative susceptibility of different monolithic materials to premature flexural fatigue, particularly in challenging prosthetic configurations such as wide unsupported anterior spans and distal cantilevers, therefore serving as indicators of potential mechanical vulnerability under repetitive loading conditions. Future studies incorporating dynamic cyclic loading protocols, thermomechanical aging, more clinically representative loading scenarios, as well as correlation with finite element analysis, are warranted to further validate and expand upon the present findings.

From a biomechanical perspective, it should be noted that the ideal prosthetic material for full-arch rehabilitations must balance sufficient structural rigidity with adequate damping capacity to dissipate loads transmitted during function. Materials with very high stiffness normally transmit greater deleterious loads to supporting implants, surrounding bone and soft tissues. While all tested high-performance polymers significantly outperformed the G3-PMMA, the linear mixed models isolated G-4 FRC and G2-PEKK as the superior materials balancing rigidity, as evaluated by the contained vertical displacements, and damping capacity. Specifically, G4-FRC demonstrated the most efficient dissipation of principal tensile forces and the lowest absolute strain peaks. Such findings are probably attributable to the multidirectional glass-fiber network, which effectively resists bending moments and limits deformation of the prostheses. Therefore, while unreinforced PAEKs provide a viable, controlled-displacement alternative for shorter spans, FRC currently presents the most optimized biomechanical balance of rigidity and energy dissipation for wide-span, monolithic rehabilitations.

## 5. Conclusions

This study demonstrates that the biomechanical behavior of polymer-based monolithic ISFPs is highly material-dependent. Milled PMMA exhibited severe flexural deformation and critical tensile strain accumulation at both the anterior unsupported span and cantilever region, whereas PEEK and PEKK showed a more favorable mechanical performance, yet presented some areas of high peak strains. FRC presented the most favorable profile, minimizing critical tensile strains in high-risk cantilever regions thus balancing rigidity and energy dissipation.

Despite the in vitro nature of the study, these findings suggest that high-performance polymers and, in particular, fiber-reinforced composites represent promising alternatives for monolithic full-arch rehabilitations.

## Figures and Tables

**Table 1:** Estimated marginal means (95%CI) of the vertical displacements, tensile (Ɛ1), compressive (Ɛ2), and von Mises (ƐVM) strains of the anterior unsupported span of the prostheses under 200 N load, as obtained by the application of the linear mixed model evaluating the full field distribution, and 99th percentile peak values.

Material	Vertical Displacement (µm)		Tensile Strain Ɛ1 (µƐ)		Compressive Strain Ɛ2 (µƐ)		von Mises Strain ƐVM (µƐ)	
	EMM [95%CI]	1st percentile peak	EMM [95%CI]	99th percentile peak	EMM [95%CI]	1st percentile peak	EMM [95%CI]	99th percentile peak
PEEK	−250.9 [−310.3, −191.5]	−413.0	1326.6 [1175.9, 1477.2]	3946.1	−1642.5 [−1968.5, −1316.5]	−10,101.3	1309.7 [1122.1, 1497.4]	6281.4
PEKK	−377.1 [−436.4, −317.7]	−669.4	1642.6 [1492, 1793.3]	5139.8	−1767 [−2093, −1441]	−8625.1	1500.1 [1312.5, 1687.8]	5875.3
PMMA	−710.2 [−769.6, −650.8]	−1013.9	2666.1 [2515.3, 2816.9]	13,503.3	−2495.1 [−2821.3, −2168.9]	−9718.2	2297.4 [2109.6, 2485.1]	8693.3
FRC	−428.9 [−488.3, −369.5]	−558.8	754.4 [603.7, 905]	4447.1	−1035.6 [−1361.6, −709.6]	−10,718.2	816.3 [628.6, 1004]	6819.9

**Table 2:** Estimated marginal means (95%CI) of the vertical displacements, tensile (Ɛ1), compressive (Ɛ2), and von Mises (ƐVM) strains of the cantilever portion of the prostheses under 200 N load, as obtained by the application of the linear mixed model evaluating the full field distribution, and 99th percentile peak values.

Material	Vertical Displacement (µm)		Tensile Strain Ɛ1 (µƐ)		Compressive Strain Ɛ2 (µƐ)		von Mises Strain ƐVM (µƐ)	
	EMM [95%CI]	1st percentile peak	EMM [95%CI]	99th percentile peak	EMM [95%CI]	1st percentile peak	EMM [95%CI]	99th percentile peak
PEEK	−279.7 [−371.5, −188.0]	−887.3	1198.4 [1080, 1316.8]	4894.3	−1206.6 [−1366.5, −1046.7]	−4256.7	1079.1 [977.3, 1180.9]	3279.8
PEKK	−217.2 [−308.9, −125.5]	−682.3	1028.9 [910.5, 1147.4]	4268.1	−722.2 [−882.2, −562.3]	−3244.2	800.3 [698.5, 902.1]	2578.7
PMMA	−562.9 [−654.7, −471.2]	−1881.7	1661.4 [1543, 1779.9]	6405.3	−2274.2 [−2434.1, −2114.2]	−6138.1	1769.5 [1667.7, 1871.3]	4203.8
FRC	−248.8 [−340.5, −157.1]	−667.4	880.6 [762.2, 999]	4686.2	−841.3 [−1001.3, −681.4]	−4578.6	784.4 [682.7, 886.2]	3427.9

## Data Availability

The original contributions presented in this study are included in the article. Further inquiries can be directed to the corresponding author.
